# Biomechanics of shear-sensitive adhesion in climbing animals: peeling, pre-tension and sliding-induced changes in interface strength

**DOI:** 10.1098/rsif.2016.0373

**Published:** 2016-09

**Authors:** David Labonte, Walter Federle

**Affiliations:** 1 Department of Engineering, University of Cambridge, Cambridge CB2 3EJ, UK; 2 Department of Zoology, University of Cambridge, Cambridge CB2 3EJ, UK

**Keywords:** controllable adhesion, biological adhesives, adhesive tapes, frictional dissipation

## Abstract

Many arthropods and small vertebrates use adhesive pads for climbing. These biological adhesives have to meet conflicting demands: attachment must be strong and reliable, yet detachment should be fast and effortless. Climbing animals can rapidly and reversibly control their pads' adhesive strength by shear forces, but the mechanisms underlying this coupling have remained unclear. Here, we show that adhesive forces of stick insect pads closely followed the predictions from tape peeling models when shear forces were small, but strongly exceeded them when shear forces were large, resulting in an approximately linear increase of adhesion with friction. Adhesion sharply increased at peel angles less than *ca* 30°, allowing a rapid switch between attachment and detachment. The departure from classic peeling theory coincided with the appearance of pad sliding, which dramatically increased the peel force via a combination of two mechanisms. First, partial sliding pre-stretched the pads, so that they were effectively stiffer upon detachment and peeled increasingly like inextensible tape. Second, pad sliding reduces the thickness of the fluid layer in the contact zone, thereby increasing the stress levels required for peeling. In combination, these effects can explain the coupling between adhesion and friction that is fundamental to adhesion control across all climbing animals. Our results highlight that control of adhesion is not solely achieved by direction-dependence and morphological anisotropy, suggesting promising new routes for the development of controllable bio-inspired adhesives.

## 1. Introduction

Many insects, spiders, lizards and tree frogs can climb on plants and in the canopy of trees by employing adhesive footpads, which allow them to switch between strong attachment and effortless detachment within fractions of a second [1–4]. The functional principles underlying this impressive dynamic control of attachment forces have attracted considerable interest among physicists, engineers and biologists, aiming to develop technical adhesives with similar properties [5]. A key feature of dynamic biological adhesive pads is that attachment forces increase when pads are pulled towards the body but decrease when pushed away from it [6–10]. This simple, reversible and fast control mechanism has a much larger influence on the pads' adhesive force than retraction speed or normal pre-load [6,8,10]. Strikingly, shear-sensitive adhesion has been reported for ‘hairy’ and ‘smooth’ as well as ‘dry’ and ‘wet’ biological adhesive pads [6–9], suggesting a universal control principle independent of pad morphology, and the alleged adhesive mechanism (van der Waals and capillary forces for dry and wet adhesives, respectively). What are the mechanisms giving rise to shear-sensitive adhesion?

**Figure 1.**
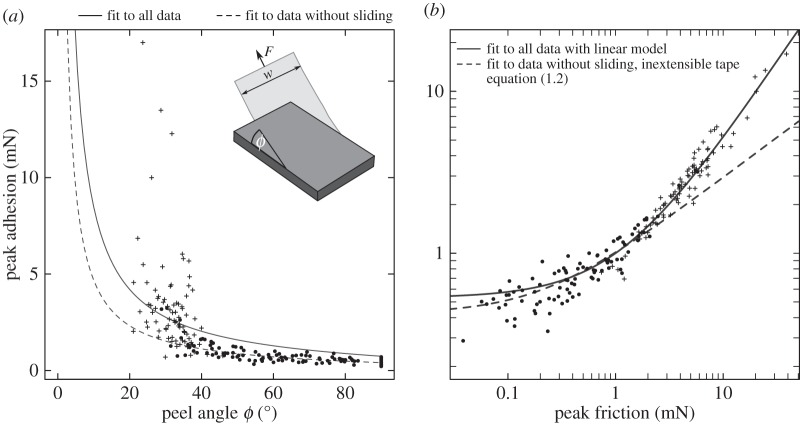
Individual pads of Indian stick insects were peeled off glass coverslips at different angles while measuring both adhesive and frictional forces. The symbols indicate whether peeling was accompanied by visible sliding (crosses for sliding or dots for static detachments, respectively). (*a*) Peak adhesion, *F*sin(*ϕ*), against peel angle (*n* = 11 insects). The inextensible tape equation systematically overestimated adhesion for large peel angles (continuous line). For peel angles smaller than ≈35°, most of the pads slid visibly during detachment. The model fit improved considerably when equation (1.2) was restricted to data from measurements where no visible sliding occurred during detachment (dashed line). (*b*) Same data as in (*a*), but on a double logarithmic scale and with friction on the *x*-axis. The predictions of a simple linear model and the inextensible tape equation are similar for small shear forces (or large peel angles), but differ increasingly for large shear forces (or small peel angles). The divergence of the two models coincides with the onset of sliding.

Several previous studies have interpreted shear-sensitive adhesion using peeling theory [6,7,11–16], which predicts the force *F* required to peel off an elastic tape of width *w*, under a peeling angle *ϕ* (see inset in figure 1*a*).

Assuming that the tape is infinitely flexible in bending, that deformations are in the limit of linear elasticity, that effects due to inertia are negligible and that peeling is in steady state,^1^ the critical peel force per unit tape width, *P* = *F*/*w*, can be linked to the tape's critical energy release rate *G* [18,19] (all equations are derived in detail in the electronic supplementary material)1.1
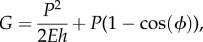
where *E* is the Young's modulus of the tape and *h* is its thickness. The first term on the right-hand side, often called ‘elastic term’, is a combination of the energies (per unit area of detached tape) required to elastically stretch the detached fraction of the tape, and to move the point of force application owing to tape stretching. The second term represents the work involved in moving the point of force application when a unit area of the tape is detached without stretching. For a thin tape of high stiffness, or for sufficiently large peel angles, equation (1.1) approximately reduces to1.2
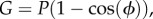
which we will refer to in the following as ‘inextensible tape model’, as it is exact for tapes of zero extensibility. Equations (1.1) and (1.2) both predict that the critical peel force, *P*, increases with the shear force, *P*cos(*ϕ*), which acts parallel to the surface. However, *P* in equation (1.1) approaches a maximum of 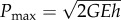 as *ϕ* → 0, whereas *P* in equation (1.2) is unbounded as a consequence of the assumption of infinite tape stiffness.

Despite the apparent differences in geometry between a simple adhesive tape and biological adhesive pads, equations (1.1) and (1.2) have been used with some success to study shear-sensitive adhesion in geckos, and tree frogs [6,7,20]. However, several problems arose. For example, the values for *G* and *E* required to fit experimental data exceeded plausible estimates, suggesting that additional dissipative mechanisms were at play [7]. In *Gekko gecko*, adhesion increased linearly with shear force, with a slope of approximately 0.5, indicating a constant ‘critical angle of detachment’ of *ca* 30° (defined as the arc tangent of the ratio between adhesion and friction, see [6]). This is in contradiction to equations (1.1) and (1.2), as the adhesive force, *F*sin(*ϕ*), cannot vary at a constant peel angle if *G* is constant. Autumn *et al*. [6] thus rejected simple tape peeling models as an explanation for shear-sensitive adhesion in geckos. Several modifications of equation (1.1) have been proposed since [11–13,21], but the mechanics of shear-sensitive adhesion in insects, tree frogs and geckos still remain unclear.

Here, we study the biomechanics of controllable adhesion in stick insects (*Carausius morosus*). This article is organized as follows. First, we show that shear-sensitive adhesion is consistent with peeling theory for large peel angles (or small shear forces), but is closer to a linear relationship between adhesion and friction for small peel angles (or large shear forces). Second, we demonstrate that the departure from peeling theory coincides with the appearance of sliding during detachment, which sometimes led to re-attachment of previously detached parts of the adhesive pads. Third, we use a simple first-principle modification of equation (1.1) to discuss how ‘pre-strain’, sliding and ‘crack-healing’ can make even soft and thin tapes behave like effectively infinitely stiff tape. Lastly, we argue that this effect is still not sufficient to fully explain the discrepancy between peeling models and observed shear-sensitive adhesion. Instead, we provide evidence for a sliding-induced increase in interface strength, and suggest that in combination, the effects of sliding can account for the linear relationship between friction and adhesion observed in biological adhesives.

## 2. Results and discussion

The critical adhesive force, *F*sin(*ϕ*), required to peel off individual adhesive pads of stick insects from glass decreased significantly with the peel angle (figure 1*a*). A nonlinear mixed model least-squares fit of the inextensible tape model, allowing us to treat different individuals as random effect, yielded a critical energy release rate of *G* = 1166 mN m^–1^ (fitted to all data, 95% CI (1023,1309) mN m^–1^), not unusual for rubbery materials, but considerably higher than expected for van der Waals forces (we justify the use of the inextensible tape equation below). This discrepancy is likely explained by viscous dissipation in the pad cuticle, as *G* approaches values typical for weak intermolecular forces in the limit of small peel velocities [10]. However, the inextensible tape model fit systematically overestimated adhesion for larger angles, and underestimated forces for smaller angles (figure 1*a*). In addition, we measured no peel angles (determined by the measured force vector) smaller than ≈22°, despite two treatments which involved smaller surface ‘retraction angles’, indicating that some pads were sliding during detachment. Indeed, high-speed recordings of the contact area during detachment revealed that 81 of 94 pads slid visibly when peeled at angles *ϕ* < 40°. When the fit of equation (1.2) was restricted to data from detachments without visible sliding (yielding *G* = 667 mN m^–1^, 95% CI (510,824) mN m^–1^), the agreement between theory and experiment considerably improved (figure 1*a*).

In contrast to the inextensible tape model, a simple linear model, *F*_A_ = *aF*_F_ + *b*, where *F*_A_ is adhesion and *F*_F_ is friction force, was in excellent agreement with the data, and explained around 95% of the overall variation in adhesion (figure 1*b*). A least-squares mixed model regression yielded a slope of 0.47 and an intercept of 0.53 mN (95% CI: (0.45,0.48) and (0.41,0.64 mN), respectively); there was no significant difference between sliding and non-sliding pads (*t*_184_ = 0.74, *p* = 0.46, *n* = 11). Adhesion was approximately half of the acting shear force, indicating a critical detachment angle of ≈30°, in remarkable agreement with earlier observations on gecko setae, despite the striking difference in pad morphology [6,22]. However, we did find significant adhesion in the absence of shear forces (i.e. for *ϕ* = 90°, *t*_186_ = 8.98, *p* < 0.001, *n* = 11, figure 1*a*), inconsistent with the phenomenological, zero-intercept ‘frictional adhesion’ model [6].

A plot of adhesion against friction on a log–log scale, along with a fit of (i) the inextensible tape equation restricted to detachment without sliding and (ii) a linear model shows that the predictions of both models are similar for small friction forces (or large peel angles; figure 1*b*). A comparison of the corresponding Akaike information criteria suggested that the inextensible tape model was in fact marginally better for friction forces smaller than approximately 2 mN (or *ϕ* > 35°; see electronic supplementary material). For friction forces larger than approximately 2 mN (or *ϕ* < 35°), however, the model predictions became increasingly different, and the linear model was more accurate. The point of divergence coincided with the onset of sliding (figure 1*b*).

### 2.1. Pre-tension, partial sliding and ‘crack-healing’

The pads' transition from static contact to sliding was likely preceded by partial slippage close to the peel front. Such interfacial slippage can lead to a dramatic increase in the apparent critical energy release rate [21,23–26], as sliding ‘consumes’ part of the available energy, so that equations (1.1) and (1.2) are no longer valid. Gravish *et al*. [27] suggested that the adhesive strength of gecko setae is largely based on ‘external’ dissipation via seta sliding, superior to many commercial soft adhesives where interface toughness is largely based on ‘internal’ dissipation via viscous deformation, compromising structural integrity and thus limiting reusability. Indeed, the thin secretion layer covering the pads of all insects studied to date may serve as a lubricating ‘release layer’, helping to reduce viscous dissipation in the pad cuticle during voluntary detachment [10].

When a fraction of the attached pad slides, it will be stretched, resulting in an increase in the system's elastic energy, and an associated movement of the point of force application. Remarkably, the energy lost to friction is of equal magnitude to the corresponding change in the elastic term arising from stretching [5,21] (and see electronic supplementary material). Upon detachment, however, the now pre-stretched pad extends less than an unstretched pad, and thus the work done by the applied load decreases. As a consequence, the required peel force increases—the interface gains strength. In this sense, peeling with frictional sliding is similar to the peeling of a tape which has been stretched *prior* to surface attachment, a case which has been thoroughly addressed by previous work [13,28–33].

**Figure 2.**
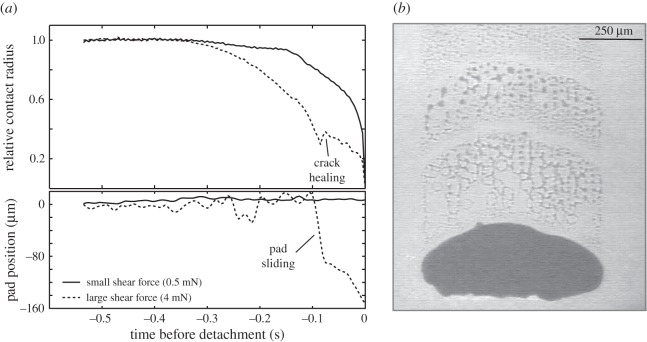
(*a*) During detachment, the contact radius of the pads—approximated as the ratio between pad area *A* and perimeter *Γ*—decreased continuously. The change of *A*/*Γ* with time may be interpreted as the speed of a crack propagating through the interface [35], which was measured by performing an ordinary least-squares regression of *A*/*Γ* against time for the last 50 ms of detachment (‘terminal’ crack speed, figure 4). When shear forces were small, the crack initially accelerated, followed by approximately steady crack growth until detachment was complete. When shear forces were large, however, we sometimes observed that the crack was arrested or even receded, i.e. detached parts of the pad re-attached (top panel). This ‘crack-healing’ was clearly associated with the onset of fast sliding, i.e. the pad's position relative to the surface changed (bottom panel). (*b*) When pads were slid across a surface, they left a trail of fluid behind.

A quantitative assessment of the effect of pad pre-tension requires an approximation of the force that pre-strained the pad. For frictional sliding, this force is *F*_0_ = *F*cos(*ϕ*) (see electronic supplementary material). Pre-tension may have arisen via one or a combination of two mechanisms in our experiments: first, low-angle peeling and frictional sliding can result in measurable strain in the pad cuticle [34]. Second, we observed ‘crack-healing’, i.e. previously detached parts of the pads reattached when peeling occurred at low angles (see [28] for similar observations on rubber tapes, and figure 2*a*). In both cases, the peel force increased further after pre-tension was induced, so that *F*_0_ = *F*cos(*ϕ*) is a plausible conservative estimate, independent of whether pads were stretched while in contact, or when detached. With this pre-strain, equation (1.1) becomes (see electronic supplementary material)2.1
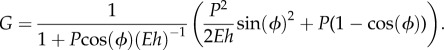
This result differs slightly from previous models for pre-strained tape [13,28–32,35], which we discuss in more detail in the electronic supplementary material.

Strikingly, a similar yet not identical result is obtained when the effect of frictional sliding (leading to pre-strain) is considered [5,21] (see electronic supplementary material)2.2
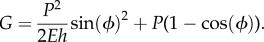
Equations (2.1) and (2.2) indicate that *identical* pre-tension at the peel front can lead to *different* peel strength if peeling is associated with interfacial slippage. This discrepancy is solely based on the fact that the peeled unit length refers to *unstretched* tape in the tape-sliding model, but to *stretched* tape in the pre-strain model (see electronic supplementary material).

**Figure 3.**
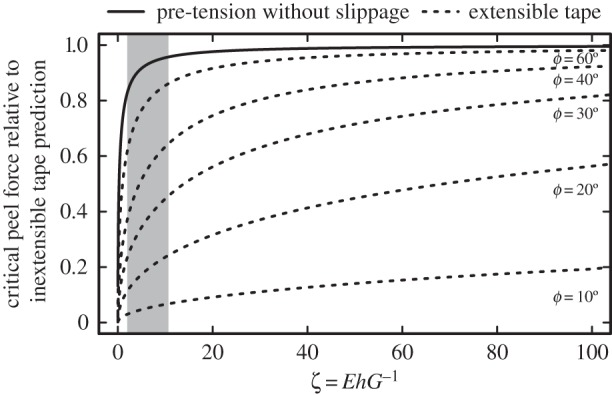
If adhesive tapes are pre-stretched with a force *F*_0_ = *F*cos(*ϕ*) and peel without partial sliding, the critical peel force is an approximately constant fraction of the force required to peel an inextensible tape with identical critical energy release rate, independent of peel angle. Even for moderately large ratios *ζ* = *EhG*^−1^ as found in biological adhesive pads (grey box, see electronic supplementary material), the peel strength is close to that of an inextensible tape. As a comparison, dashed lines show the critical peel force for an extensible tape relative to the inextensible tape equation for different peel angles.

The difference between equations (2.1) and (2.2) is governed by a dimensionless parameter, *ζ* = *Eh*/*G*, which may be interpreted as the ratio of the elastic and adhesive work during peeling (see electronic supplementary material). The two models are increasingly similar for large values of *ζ*, as both approach the inextensible tape model (equation (1.2)) as *ζ* → ∞. However, even moderately large values of *ζ* can lead to effectively inextensible behaviour. This can be illustrated with a simplified version of equation (2.1), which can be found by assuming that the change in the surface energy term owing to the additional peeled length arising from pre-tension is negligible (see electronic supplementary material):2.3
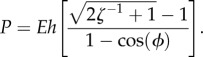
Equation (2.3) can be used for the peeling of a pre-strained tape in the absence of interfacial slippage, and is identical to the result given by [13,28,29,31–33] for *F*_0_ = *F*cos(*ϕ*). Although incorrect, equation (2.3) sets a conservative limit, and is reasonably close to the exact solution for large peel angles and *ζ* > 1 (see electronic supplementary material), which is likely the case for most technical tapes and biological adhesives (see figure 3). The ratio of this force to the critical peel force for an inextensible tape is independent of the peel angle, and solely determined by *ζ*, i.e. adhesion tends to infinity as *ϕ* → 0. For a thin and soft tape with *G* = 100 m Nm^−1^, *h* = 100 µm and *E* = 1 MPa, we find *ζ* = 10^3^, and the prediction of equation (2.3) is within 0.05% of the inextensible tape model (equation (1.2), figure 3). Even for very soft and thin structures such as stick insect pads (with ζ > 5), the agreement is within 10% (figure 3). In practice, however, the yield strength of the tape may limit the force-enhancing effect of pre-tension considerably, and for elastomers, strains may be sufficiently large to invalidate small-strain approximations. Models for large strains can be found in [21,33].

Partial sliding during peeling will decrease the peel force in comparison with a tape with identical pre-strain at the peel front peeled without partial sliding. However, even with partial sliding, the peeling behaviour is similar to that of inextensible tape if *ζ* > 100 (see electronic supplementary material). While the critical peel force for peeling with partial sliding is unbounded as the peel angle approaches zero, the adhesive force per unit tape width, *P*sin(*ϕ*), remains finite and approaches 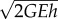. As *ϕ* → 0, an increasing fraction of the peel force acts in shear and is lost to sliding [21]. Hence, the critical energy required for crack propagation must be supplied by normal stresses, so that the *adhesive force* of peeling with sliding is approximately the same as the *total peel force* for an extensible adhesive tape peeled without sliding (see equation (1.1)). When peeled at 0°, insufficient energy would be left to drive the crack through the interface, so that the pads would merely slide [21].

The enhancing effect of pad pre-tension, both with and without interfacial slippage, readily justifies the use of the inextensible tape model even for small peel angles (figure 3). The effect of pre-tension may also explain why a fit of the extensible tape model (equation (1.1)) to data from tree frogs required unrealistically large values for *E* [7]: owing to the pre-tension-induced changes in the peeling energy balance, even soft and thin pads can behave like an inextensible tape (figure 3). This considerable increase in apparent stiffness during low-angle peeling is likely of major importance not only for the shear-sensitivity of smooth pads in stick insects, but for dynamic biological adhesives in general.

### 2.2. Increase of the critical energy release rate via pad sliding

Pad pre-tension can result in a significant increase of the critical peel force in comparison with an unstretched pad, but it remains unclear whether it can fully account for shear-sensitive adhesion as observed in geckos, tree frogs and stick insects. Notably, pre-tension of an adhesive tape prior to peeling can lead to a critical detachment angle [13], seemingly consistent with observations on gecko adhesion [6,22], and our data on stick insects. However, while all the modified peel models, including those for pre-strain and partial sliding (equations (2.3) and (2.4)) predict *smaller* forces than the inextensible tape model (equation (1.2); electronic supplementary material), the adhesion forces measured at low peel angles strongly *exceeded* this prediction (figure 1). This discrepancy is far from trivial: for a feedback-maintained shear force of 10 mN, we measured an adhesive force of 4.08 ± 0.87 mN (*n* = 11), corresponding to approximately two-thirds of the insect's body weight. Achieving the same level of adhesion with an inextensible tape of the estimated critical energy release rate would require a friction force of 18 mN, or three times the body weight. The adhesion forces predicted by the inextensible tape model (equation (1.2)) scale with the square root of the applied friction force for friction forces much larger than *Gw* [9]. However, we found an approximately linear relationship, i.e. the observed attachment forces (for large friction forces) exceeded the inextensible tape prediction by a factor approximately proportional to the square-root of the applied friction. In addition, a critical detachment angle as predicted by [13] does not occur if pad pre-stretch is based on sliding. Clearly, the shear sensitivity of adhesion and the apparent critical detachment angle cannot be explained by any of the simple peeling models accounting for pre-tension and/or partial sliding. How then do sliding pads achieve forces much higher than the prediction of the inextensible tape model? Our findings on insects, and earlier results on geckos [6] can only be reconciled with peeling theory if the critical energy release rate *G* increases with the applied friction force (see equation (1.2)).

In fracture mechanics, *G* is often modelled as a function of ‘mode-mixity’, i.e. the extent to which interfacial failure occurs via shear versus tensile stresses [30,36,37]. For tape peeling, however, the mode-mixity dependence of *G* may largely arise from frictional ‘dissipation’ [21,26], and is thus unlikely to provide an explanation (see above). Instead, the ‘true’ tensile strength of the interface must increase. Kendall [28] observed that crack-healing in rubber tapes peeled at low angles was accompanied by a significant increase in the critical energy release rate measured for *receding* cracks. Kendall suggested that this ‘surface activation’ may be partly explained by triboelectric charging, and indeed, sliding during tape peeling can lead to significant charges at the interface [38]. In order to test whether triboelectric charging can explain the observed increase of *G* for smaller peel angles, we repeated our experiments on grounded glass coverslips coated with conducting indium tin oxide (ITO). The relationship between peel angle and adhesion was virtually identical (see the electronic supplementary material). We therefore conclude that even if present, surface charging did not lead to a significant increase of the critical energy release rate *G*.

**Figure 4.**
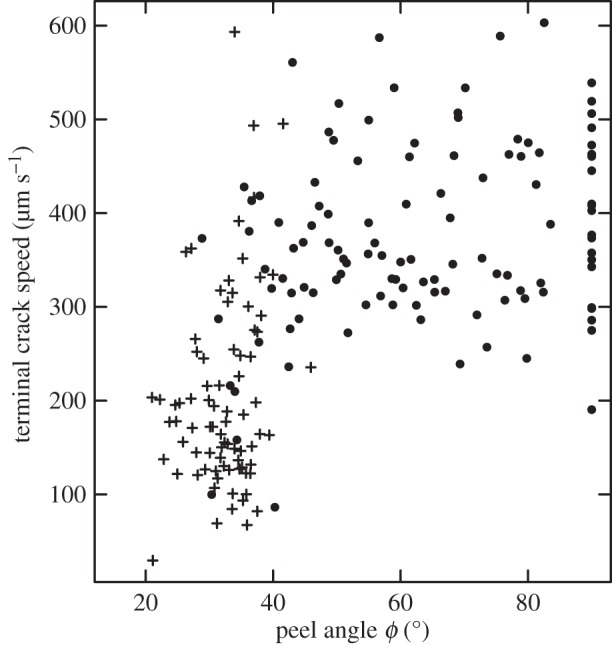
As a result of pad sliding and associated crack-healing (figure 2*a*), detachments with visible sliding (crosses) exhibited a systematic decrease in crack propagation speed for peel angles smaller than ≈35°. Dots represent detachments without visible sliding.

Adhesion depends on the ability of the interface to sustain stress. Insect adhesive pads are covered with a thin film of a secretion that acts as a separation layer, allowing minimization of viscoelastic losses during rapid detachments by providing a highly mobile interface through which a crack can easily propagate [10]. This effect, akin to slippage, reduces the critical stress concentration required for crack propagation, so that detachment forces remain small during voluntary detachment (see [39–41] for examples on synthetic adhesives). Pad sliding is accompanied by a loss of pad secretion at the pad's trailing edge (figure 2*b*), which leads to a significant increase in shear stress [42–45]. A higher shear stress in a soft material implies an increase in adhesion hysteresis [46,47], and hence an increase of *G* upon reduction of the secretion film thickness (see also [48]). Sliding-induced fluid squeeze-out and an associated increase in interfacial strength have also been observed for rubber blocks lubricated by grease or glycerol, but was absent when a stiff material was used instead of the rubber [49]. An increase in *G* as a result of sliding is also consistent with the lower crack propagation speed measured for sliding pads, despite higher or equivalent normal stresses (figure 4) [50]. As the rate dependence of friction and bulk dissipation can differ considerably, even a minor increase in interfacial friction can change the adhesive force substantially [50]. An increase in *G* triggered by sliding may also be plausible for gecko setae [27], which have been shown to leave phospholipid footprints behind [51]; these could fulfil a similar function to the pad secretion in arthropods. Clearly, further research is required to investigate the role of interfacial mobility in biological adhesives.

Interface strengthening via sliding has at least two biologically relevant advantages over a typical peeling situation. First, as the onset of sliding depends on the pad's contact area, not only friction, but also adhesion forces may scale with pad area, which is increasingly difficult to achieve for larger animals [9]. Area scaling of adhesion is consistent with the observed, approximately linear relationship between friction and adhesion, and may be mediated by the pre-stretching of the pad, leading to a more uniform stress distribution across the pad contact zone. Second, the increase in *G* with friction force effectively expands the range of peel angles for which strong attachment is possible, but adhesive strength vanishes quickly when peel angles are larger than 30°, allowing a rapid switch between strong attachment, and effortless detachment during locomotion (by a minimal change of the direction of the force vector, see [6,13]).

## 3. Conclusion

We have shown that shear-sensitive adhesion in insects is consistent with classic peeling theory if friction forces are small, but a linear relationship between friction and adhesion occurs when friction forces are large. This coupling between adhesion and friction leads to a sharp increase of adhesion at peel angles smaller than 30°, which may result from two effects of sliding. First, partial sliding during detachment can give rise to considerable pre-tension, so that the pads have an increased apparent stiffness. Second, the thin films formed by the pad secretion result in a coupling of interfacial and bulk properties: pad sliding reduces the thickness of the fluid layer in the contact zone, and the interface now has a lower mobility, so that slippage is reduced, and stresses need to rise to higher levels to drive detachment. Larger stress levels increase the deformed volume of the adhesive pad, thereby increasing bulk dissipation within the adhesive pad cuticle [25]. As a consequence, peel forces exceed the predictions for an inextensible tape with constant critical energy release rate *G*. However, previous experiments on insects have shown that the influence of shear forces on adhesive performance is much larger than the influence of retraction speed, suggesting that the role of viscoelastic dissipation may be small [10]. In combination, these effects may explain the sharp increase of adhesion with decreasing peel angle, and the approximately linear relationship between adhesion and friction observed in dynamic biological adhesives, allowing climbing animals to switch rapidly between attachment and detachment.

Our results demonstrate that the impressive controllability of biological adhesives does not solely arise from the pads' morphology, structural anisotropy and direction-dependence, but is directly linked to processes at the interface. This finding suggests a promising new route for the development of highly controllable bio-inspired adhesives with a simple morphology. Most technical adhesives are polymers, whose interfacial properties can be fine-tuned on a molecular scale. The extensive theoretical and experimental toolbox available for studying and modifying polymers [50,52] should allow the creation of technical adhesives that reversibly increase in strength when slid, replicating one of the most desirable and defining features of biological adhesives.

## 4. Material and methods

**Figure 5.**
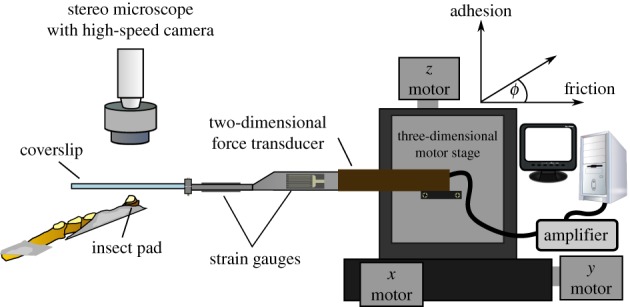
Experimental set-up for measuring adhesion, friction and contact area of single attachment pads. (Online version in colour.)

Attachment performance of single pads of live Indian stick insects (*Carausius morosus*, Sinéty, 1901; mass = 618 ± 101 mg, mean ± s.d., *n* = 11) was measured with a custom-made two-dimensional-force transducer set-up described in detail in Drechsler & Federle [43] (see figure 5 for a schematic of the set-up). The pads were mounted using the method described in Labonte & Federle [8]. During the force measurements, the contact area of the pads was recorded with a Redlake PCI 1000 B/W high-speed camera (Redlake MASD LLC, San Diego, CA), mounted on a coaxially illuminated stereo-microscope (Leica MZ16, Leica Microsystems GmbH, Wetzlar, Germany). All measurements were conducted at 22–24°C and 40–50% humidity, and with clean glass or ITO-coated coverslips purchased from Diamond Coatings Ltd (Halesowen, UK). The ITO coverslips had a resistance of 15–30 Ω, measured with electrodes attached on opposite sides of the 18 × 18 mm coverslips (Fluke 27 multimeter, RS Components Ltd, Corby, UK); the coverslips were grounded during the force measurements.

### 4.1. Measurement protocol

Peak adhesion of stick insect arolia was measured under two different conditions for all specimens (i) by retracting the coverslips with constant speed and different constant ‘retraction angles', altered by adjusting the movement velocity of each motor axis and (ii) by retracting the coverslips perpendicularly after a defined shear force was applied to the pads [8]. The order of the conditions was randomized, and each measurement was performed on a fresh position of the surface, in order to avoid a systematic influence of fluid accumulation and/or depletion [43,44].

For the first measurement series, the surface was initially pressed onto the pads with a normal pre-load of 1 mN, corresponding to approximately one-sixth of the body weight, controlled via a motorized 20 Hz force-feedback mechanism incorporated in a custom-made LabVIEW control software (National Instruments, Austin, TX). After a contact time of 5 s, the surface was retracted at a defined retraction angle (given by the motor trajectory), with a constant motor speed along the trajectory of 0.5 mm s^–1^. Measurements were performed for nine retraction angles, ranging from 90° to 10° in steps of 10° (here, 90° corresponds to a perpendicular pull-off). For the second series of measurements, the surface was pressed onto the pads with a pre-load of 1 mN for 5 s as before. Subsequently, the motorized force-feedback mechanism was used to apply a constant shear force for a period of 3 s, followed by a perpendicular pull-off at 0.5 mm s^–1^ (i.e. during detachment, the beam was only moved perpendicularly by the motors). Measurements were performed for eight different shear forces, ranging from 5% to 170% of the body weight (0.25, 0.5, 1, 2, 4, 6, 8 and 10 mN).

Force–displacement data were recorded with an acquisition frequency of 20 Hz, and the pad contact area was filmed at 200 frames per second for shear-force feedback experiments, or at 100 frames per second for the measurements that involved detachment at defined retraction angles. The difference in frame rate was because of the limited memory of the camera and the longer times required to detach the pads at peeling angles less than 30°.

For both types of measurements, peak adhesion and the friction forces at this peak were extracted from the force–time curves. From these data, we also calculated the peel angle, i.e. the arc tangent of the ratio of both forces. As the relationship between peel angle and adhesion did not differ between the two types of experiments, the data were pooled (repeated-measures ANCOVA, *F*_1,192_ = 2.39, *p* = 0.12, *n* = 11 for both types of measurements).

In order to measure the width *w*, area *A*, and perimeter *Γ* of the pad contact area, the video recordings were post-processed using Fiji [53]. Video recordings were filtered in order to remove flickering from the light source and subsequently converted into binary images using the fuzzy threshold algorithm described in [54]. The binary images were despeckled using 2 × 2–5 × 5 pixels median filters and the resulting stacks were analysed with the native particle analysis routines implemented in ImageJ 1.48 k.

From the processed videos, we also measured the speed of crack propagation *v*_c_ [35]4.1
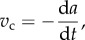
where *a* = *A*/*Γ* is the contact radius. The speed of crack propagation *v*_c_ changed during detachment, and we measured the ‘terminal’ speed of crack propagation by performing an ordinary least-squares regression of contact radius against time for the last 50 ms of detachment (i.e. for five and 10 data points for 100 and 200 Hz recordings, respectively). From the video recordings, we also determined whether pads were sliding during detachment, which was clearly visible as a change of the pad position relative to features on the coverslips. All statistical analyses were carried out with R v. 3.0.2 [55].

## Supplementary Material

Additional data, statistical tables, and detailed derivations

## Endnote

1 In animal adhesive pads, the contact zone has a finite length, so that peeling may not be steady-state and depend on the pad's bending stiffness; such factors have been considered by computational models of adhesive hairs [15,17].

## Data accessibility

All data are available from the Dryad digital repository (http://dx.doi.org/10.5061/dryad.gt78n).
