# Supplementary material for: Biomechanics of shear-sensitive adhesion in climbing animals: peeling, pre-tension and sliding-induced changes in interface strength
Source: J R Soc Interface. 2016 Sep;13(122):20160373. doi: 10.1098/rsif.2016.0373 (PMC5046945; doi:10.1098/rsif.2016.0373)
Supplement: Additional data, statistical tables, and detailed derivations [file rsif20160373supp1.pdf]

1 Shear-sensitive adhesion on conductive coverslips

We conducted the measurements described in the main manuscript on glass coverslips coated with indium tin oxide (ITO) in order to investigate whether triboelectric charging can explain part of the increase in the energy release rate at low peel angles. A repeated measures ANCOVA revealed that the relationship between adhesion and peel angle was not significantly affected by the conductivity of the surface ($F_{1,352}$=0.72, p=0.4), providing evidence that triboelectric charging is not of significant importance for shear-sensitive adhesion in insect pads (see fig. S1).

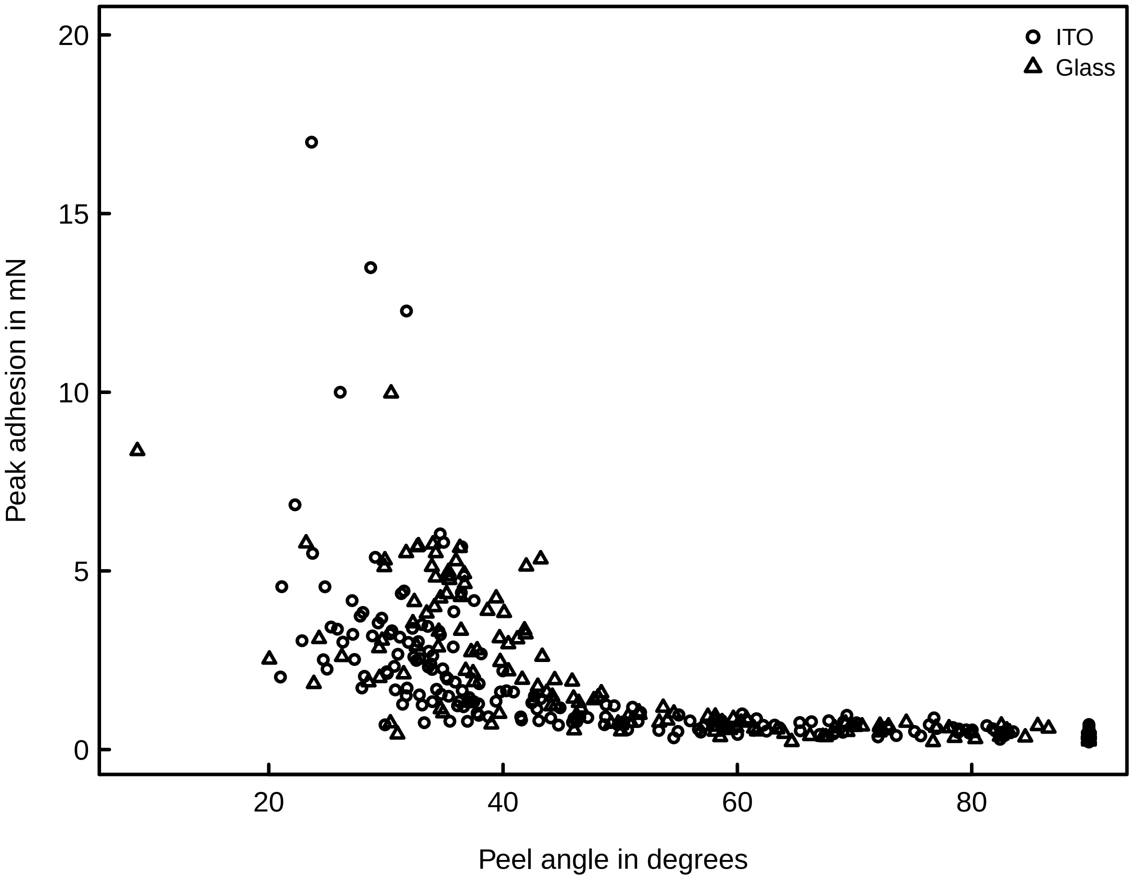

Figure S1 | The conductivity of glass coverslips did not significantly influence the relationship between adhesion and friction, suggesting that triboelectric charging does not significantly contribute to the coupling between adhesion and friction. The two lines are the result of a linear mixed model least-squares regression (n=9 and n=11 insects measured on indium tin oxide (ITO) and glass, respectively). The star indicates a measurement on ITO which was not considered for the statistical comparison in order to meet the assumptions of the linear model. Including the value did not change the principal result of the statistical comparison between measurements on ITO and glass.

2 Derivation of peel models

Here, we briefly derive the different peel models relevant for the discussion in the main manuscript, as the direct comparison helps to reveal the sometimes subtle differences between the models. Apart from our solution for the pre-strained tape, all equations have been derived previously, and we refer the reader to the corresponding work for a more detailed discussion of the models.

2.1 Inextensible tape

Imagine the situation shown in Fig. S2 A, where a thin strip of an adhesive tape of width w, and energy release rate G is subjected to a peel force F, acting with an angle ϕ to the surface. We assume that the length and stiffness of the tape are infinite, that its bending stiffness is negligible, and that peeling is in steady-state. Peeling a length L_0 requires to overcome the adhesive energy

$$W_{adh} = wL_0G \tag{S1}$$

At the same time, the tape moves along the direction of the applied force, and the corresponding work is

$$W_{pot} = Fa_0 = FL_0[1 - cos(\phi)] \tag{S2}$$

$W_{pot} = W_{adh}$ yields

$$G = P[1 - cos(\phi)] \tag{S3}$$

where $P = F/w$. Equation (S3) has first been derived by Rivlin (1944).

2.2 Extensible tape

Now consider the situation shown in fig. S2 B, which is identical to the previous case, but the tape now has a finite stiffness E. The adhesive work term remains identical, but upon detachment, the tape is stretched by the applied force, which leads to an additional movement of length a_ε along the direction of the applied force. This length is

$$a_\varepsilon = \varepsilon L_0 = \frac{\sigma}{E}L_0 = \frac{F}{Ehw}L_0 \tag{S4}$$

where h is the thickness of the adhesive tape, which experiences a stress σ. The work done by the applied load is

$$W_{pot} = F(a_0 + a_\varepsilon) = FL_0\left([1 - cos(\phi)] + \frac{F}{Ehw}\right) \tag{S5}$$

In addition, elastic energy is stored in the detached fraction of the tape

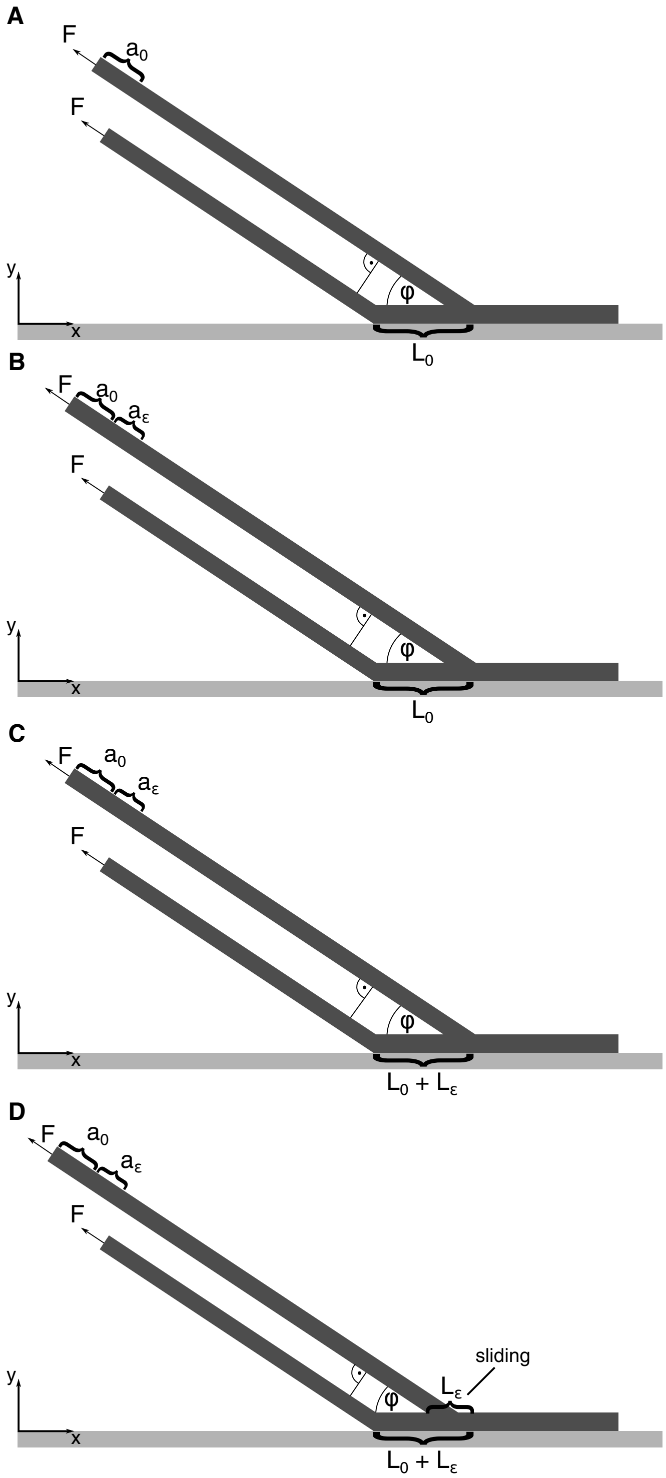

Figure S2 | Schematic drawings of various peel situations relevant for the discussion in the main manuscript. (A) Inextensible tape, (B) Extensible tape, (C) Pre-strained tape, (D) Peeling with sliding.

$$W_{elast} = \int_0^{a_\varepsilon} F d\delta = \frac{F^2 L_0}{2Ehw} \tag{S6}$$

where δ is the displacement.

With these additional terms, $W_{pot} = W_{adh} + W_{elast}$ yields

$$G = \frac{P^2}{2Eh} + P(1 - cos(\phi)) \tag{S7}$$

Equation (S7) has first been derived by Kendall (1975).

2.3 Pre-strained tape

We now assume that the tape in fig. S2 B has been stretched by a force F_0 *prior* to attachment, resulting in a 'pre-strain' $\varepsilon_0 = \frac{F_0}{Ehw}$. Analogous to the previous procedure, and noting that the peeled length is $L_0 + L_\varepsilon$ (fig. S2 C), the associated energy changes are

$$W_{adh} = wL_0 \left(1 + \frac{F_0}{Ehw}\right) G \tag{S8}$$

$$W_{pot} = FL_0 \left[\left(1 + \frac{F_0}{Ehw}\right)[1 - cos(\phi)] + \frac{F - F_0}{Ehw}\right] \tag{S9}$$

$$W_{elast} = \frac{(F^2 - F_0^2)L_0}{2Ehw} \tag{S10}$$

An energy balance yields the peel equation for a pre-strained tape

$$G = \frac{1}{1 + P_0(Eh)^{-1}} \left(\frac{(P - P_0)^2}{2Eh}\right) + P(1 - cos(\phi)) \tag{S11}$$

This result differs from previous work, which assumed that the peeled length is only L_0 (Maugis & Barquins, 1978; Williams, 1993; Williams & Kauzlarich, 2004; Molinari & Ravichandran, 2008; Chen et al., 2009). As a consequence, our solution has an additional term $(1 + P_0/(Eh))^{-1}$. Equation (S11) simplifies considerably if P_0 is assumed to be a constant fraction of the peel force, $P_0 = Pk$. Using $k = cos(\phi)$ (see main manuscript) yields

$$G = \frac{1}{1 + Pcos(\phi)(Eh)^{-1}} \left[\frac{P^2}{2Eh} sin(\phi)^2 + P(1 - cos(\phi))\right] \tag{S12}$$

Next, we note that the difference introduced by the additional term $(1 + P_0/(Eh))^{-1}$ in eq. (S11) is marginal if (i) $k = cos(\phi)$, and (ii) if the ratio of elastic to adhesive work during peeling is reasonably large [1] (see fig. S3). We thus continue in our analysis with the simplified version presented by previous authors (i. e. eq. (S11) without $(1 + P_0/(Eh))^{-1}$; Williams,

[1] Some simple manipulation is sufficient to show that the changes in P due to the prefactor in eq. (S12) are dependent on $G/(Eh)$

1993; Williams & Kauzlarich, 2004; Molinari & Ravichandran, 2008; Chen et al., 2009). As before, we use $k = cos(\phi)$, and solve for P

$$P = Eh\frac{\sqrt{2G(Eh)^{-1}+1}-1}{1-cos(\phi)} \tag{S13}$$

This result can be compared to the critical force required to peel an inextensible tape: dividing eq. (S13) by eq. (S3) solved for P, yields a ratio ν:

$$\nu = \frac{P_{pre-strain}}{P_{inextensible}} = \zeta\left[\sqrt{1+2\zeta^{-1}}-1\right] \tag{S14}$$

which is a function of $\zeta = (Eh)G^{-1}$, and is independent of the peel-angle ϕ (see main manuscript).

2.4 Extensible tape with partial sliding

Lastly, we consider the case when peeling is accompanied by sliding of a fraction of the attached tape (fig. S2 D). A horizontal pull of magnitude $Fcos(\phi)$ will exceed the static shear stress of the tape in an area close to the peel front, and thereby stretch a fraction of the tape *prior* to detachment. The length of the stretched region is

$$s = \frac{Fcos(\phi)}{\tau w} \tag{S15}$$

where τ is the average shear stress in the stretched region, which we assume to be independent of sliding velocity (the final solution is independent of the assumed relationship between shear stress and sliding velocity, see Newby & Chaudhury, 1998). At a distance y_0 inward from the peeling edge, the tape strain is

$$\varepsilon_0 = \frac{Fcos(\phi)-y_0 w\tau}{Ehw} \tag{S16}$$

which indicates a linear increase in strain from 0 at $y_0 = s$ to $Fcos(\phi)/(Ehw)$ at the peeling edge. The length of the stretched region *prior* to stretching is

$$s_0 = \frac{2EhFcos(\phi)}{\tau_0(Fcos(\phi)+2Ehw)} \tag{S17}$$

which can be found from the mean strain, $\hat{\varepsilon} = Fcos(\phi)/(2Ehw) = (s-s_0)/s_0$.

During peeling, a section of unstretched length L_0 will be stretched. During this process, the section will slide over a distance $d = s - s_0$, and it has a mean length $L_0(1+\hat{\varepsilon})$

$$d = \frac{Fcos(\phi)}{\tau_0 w + \frac{2\tau_0 Ehw^2}{Fcos(\phi)}} \tag{S18}$$

The energy lost to sliding is

$$W_{slid} = \tau_0 w L_0 (1+\hat{\varepsilon}) d = L_0 \left(\frac{F^2 cos^2(\phi)}{2Ehw} \right) \tag{S19}$$

which is independent of τ, and identical to the elastic energy required to stretch the length L_0 of the unstrained tape with a force of $Fcos(\phi)$ (see also Newby & Chaudhury, 1998; Jagota & Hui, 2011; Begley et al., 2013).

The additional relevant energy terms are

$$W_{adh} = wL_0(1 + \frac{Fcos(\phi)}{Ehw} - \underbrace{\frac{Fcos(\phi)}{Ehw}}_{Sliding})G \tag{S20}$$

$$W_{pot} = FL_0 \left[\left(1 + \frac{Fcos(\phi)}{Ehw}\right)[1-cos(\phi)] + \frac{F - Fcos(\phi)}{Ehw} + \underbrace{\frac{Fcos^2(\phi)}{Ehw}}_{Sliding} \right] \tag{S21}$$

$$W_{elast} = \frac{\left(F^2 - (Fcos(\phi))^2\right) L_0}{2Ehw} + \underbrace{L_0 \frac{F^2 cos^2(\phi)}{2Ehw}}_{Sliding} \tag{S22}$$

where we labelled the terms that arise from sliding. It is straightforward to check that these additional terms in W_{pot} and W_{elast} exactly balance W_{slid}, and only a small difference to equations 8-11 arises from the additional 'sliding' term in W_{adh} (eq. S20). An energy balance yields

$$G = \frac{P^2}{2Eh} sin^2(\phi) + P(1 - cos(\phi)) \tag{S23}$$

Equation (S23) has first been derived by Jagota & Hui (2011), and is almost identical to eq. (S12), which is valid for a tape pre-stretched with $Fcos(\phi)$, but without sliding. The only difference arises from the additional movement of the peel front to the left due to tape sliding, which alters the net change in adhesive energy associated with incremental peeling.

The different models are compared for a range of values of ζ in fig. S3. As expected, all models approach the inextensible tape model (eq. (S3)) as $\zeta \to \infty$. However, both pre-tension models and the model for sliding approach eq. (S3) much faster than a merely extensible tape. The difference between eqs. (S3), (S12) and (S13) is only relevant for small values of ζ, i.e. for softer or stickier tapes. Equation (S23) generally requires larger values of ζ in order to approach the inextensible tape equation, but the critical peel force is still significantly increased in comparison to an extensible tape.

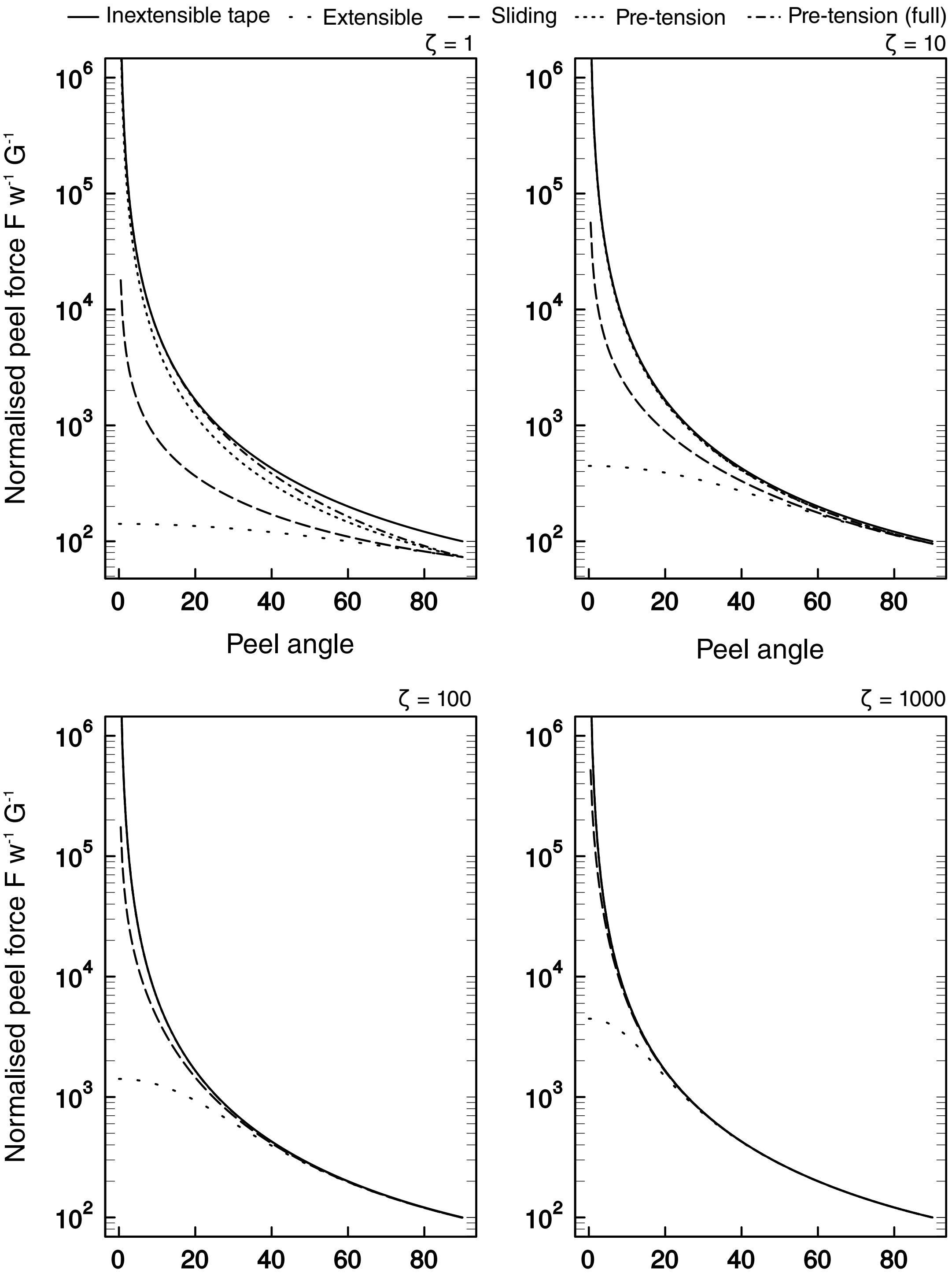

Figure S3 | Comparison of the different peel models for different values of $\zeta = (Eh)G^{-1}$.

3 Model comparison for small shear forces

We briefly compare the quality of a simple linear relationship between friction and adhesion, and the inextensible tape model re-written in terms of friction force (see Labonte & Federle, 2015), for detachments without pad sliding, and peel angles larger than 35 °. Both models have an R^2 of 0.93, but the Akaike information criterion of the inextensible tape model fit was slightly lower, because only one instead of two parameters need to be estimated from the data (the width of the pads, w, was measured from the video recordings, see tab. 1). Thus, the inextensible tape model is the superior for large peeling angles.

Table 1 | Results for mixed model fits for the inextensible tape equation and a linear relationship between adhesion, F_A, and friction, F_F, (both in mN), both restricted to peel angles ϕ larger than 35 °. In both cases, individual specimen were treated as random effects. The width of the adhesive pads w (in µm) was measured from the video recordings.

Inextensible tape, $F_A = \sqrt{wG(wG + 2F_F)}$					
Random effects	Variance				
Individual in N^2	2.06E-08				
Residual in N^2	7.71E-03				
Fixed effects	Estimate	Std. Error	t		
Strain energy release rate, G in N m^{-1}	0.66	0.045	14.56		
AIC	BIC	logLik	R^2		
-185	-177	96	0.93		
Linear model, $F_A = aF_F + b$					
Random effects	Intercept	Residual			
Individual in mN	0.101	0.0884			
Fixed effects	Estimate	Std. Error	DF	t	p
Intercept, b, in mN	0.43	0.032	97	13.27	<0.001
Slope, a	0.56	0.017	97	32.02	<0.001
AIC	BIC	logLik	R^2		
-173	-162	91	0.93		

109 Observations from 11 Individuals

4 The ratio of adhesive to elastic strength for biological adhesives

The ratio of adhesive to elastic strength, $\zeta = (Eh)G^{-1}$, determines the interfacial strength of adhesives that peel like thin strips of adhesive tape (see fig. S3). For biological adhesives, which are often thin and/or soft, a rough estimate can be obtained by using estimates for G, h, and E from the literature. For gecko spatulae, $h \approx 5$ nm, and $E \approx 1$ GPa (Persson & Gorb, 2003; Autumn et al., 2006), for spatulate tips in beetles, $h \approx 500$ nm, and $E \approx 2$ MPa (Eimüller et al., 2008; Peisker et al., 2013), and for stick insects, $h \approx 60$ µm (Scholz et al., 2008), and $E \approx 100$ kPa (A Birn-Jeffery, personal communication). Assuming that $G \approx 500$ mN m^{-1} (see main manuscript and Gravish et al., 2008), this yields $2 < \zeta < 12$.

References

Autumn K, Majidi C, Groff R, Dittmore A & Fearing R (*2006*): *Effective elastic modulus of isolated gecko setal arrays*. J Exp Biol *209*: 3558–3568.

Begley M. R, Collino R, Israelachvili J. N & McMeeking R. M (*2013*): *Peeling of a tape with large deformations and frictional sliding*. J Mech Phys Solids *61*: 1265–79.

Chen B, Wu P & Gao H (*2009*): *Pre-tension generates strongly reversible adhesion of a spatula pad on substrate*. J R Soc Interface *6*(35): 529–537.

Eimüller T, Guttmann P & Gorb S. N (*2008*): *Terminal contact elements of insect attachment devices studied by transmission X-ray microscopy*. J Exp Biol *211*(12): 1958–1963.

Gravish N, Wilkinson M & Autumn K (*2008*): *Frictional and elastic energy in gecko adhesive detachment*. J R Soc Interface *5*(20): 339–348.

Jagota A & Hui C (*2011*): *Adhesion, friction, and compliance of bio-mimetic and bio-inspired structured interfaces*. Mat Sci Eng R *72*(12): 253–292.

Kendall K (*1975*): *Thin-film peeling – the elastic term*. J Phys D: Appl Phys *8*: 1449–1452.

Labonte D & Federle W (*2015*): *Scaling and biomechanics of surface attachment in climbing animals*. Phil Trans R Soc B *370*(1661): 20140 027.

Maugis D & Barquins M (*1978*): *Fracture mechanics and the adherence of viscoelastic bodies*. J Phys D: Appl Phys *11*(14): 1989–2023.

Molinari A & Ravichandran G (*2008*): *Peeling of elastic tapes: effects of large deformations, pre-straining, and of a peel-zone model*. J Adhesion *84*(12): 961–995.

Newby B & Chaudhury M (*1998*): *Friction in adhesion*. Langmuir *14*(17): 4865–4872.

Peisker H, Michels J & Gorb S. N (*2013*): *Evidence for a material gradient in the adhesive tarsal setae of the ladybird beetle* Coccinella septempunctata. Nat Commun *4*: 1661.

Persson B & Gorb S (*2003*): *The effect of surface roughness on the adhesion of elastic plates with application to biological systems*. The Journal of chemical physics *119*: 11 437–.

Rivlin R (*1944*): *The effective work of adhesion*. Paint Technol *9*: 215–216.

Sauer R. A (*2011*): *The peeling behavior of thin films with finite bending stiffness and the implications on gecko adhesion*. J Adhesion *87*(7-8): 624–643.

Sauer R. A (*2014*): *Advances in the computational modeling of the gecko adhesion mechanism*. J Adhes Sci Technol *28*(3-4): 240–255.

Scholz I, Baumgartner W & Federle W (*2008*): *Micromechanics of smooth adhesive organs in stick insects: pads are mechanically anisotropic and softer towards the adhesive surface*. J Comp Physiol A *194*(4): 373–384.

Williams J (*1993*): *A review of the determination of energy release rates for strips in tension and bending. Part I-static solutions*. J Strain Anal Eng *28*(4): 237–246.

Williams J. A & Kauzlarich J. J (*2004*): *Peeling shear and cleavage failure due to tape prestrain*. J Adhesion *80*(5): 433–458.
